# Effectiveness of Endovascular Treatment in Native Hemodialysis Fistula Dysfunction: Long-Term Outcomes

**DOI:** 10.3390/jcm14124382

**Published:** 2025-06-19

**Authors:** Mehmet Beyazal, Esat Kaba

**Affiliations:** Faculty of Medicine Training and Research Hospital, Recep Tayyip Erdoğan University, 53020 Rize, Turkey; esatkaba04@gmail.com

**Keywords:** hemodialysis, AVF, endovascular treatment, angioplasty, patency

## Abstract

**Objectives:** This study aimed to present our single-center experience on the efficacy of endovascular treatment for the dysfunction of hemodialysis arteriovenous fistulas (AVF). **Methods:** This retrospective study analyzed 110 patients with hemodialysis AVF dysfunction who underwent endovascular treatment. Patients were evaluated with Doppler ultrasound, and those with significant stenosis or thrombosis were treated using balloon angioplasty, tissue plasminogen activator (t-PA), and/or thrombectomy, or a combination of both. A transvenous approach was performed in all cases, and post-procedural patency was assessed with fistulography. The primary outcome was defined as achieving stenosis reduction below 30%, with follow-up patency recorded at 3 and 6 months. Long-term fistula patency times and the frequency of repeat interventions were also evaluated. Statistical analysis was conducted to evaluate patency outcomes and procedural success rates. **Results:** Primary patency was achieved in 90.9% of patients, with balloon angioplasty significantly improving patency rates (*p* = 0.0077), while t-PA and thrombectomy showed no significant impact. At the 3-month follow-up, 83% of patients maintained patency; at 6 months, this rate decreased to 72.7%. ANOVA analysis showed no significant differences between treatment groups in long-term patency time (*p* = 0.322). The mean fistula patency duration was most prolonged in patients treated with balloon angioplasty alone (21.8 months), followed by those who received combination therapy (19.2 months), and shortest in those treated with only t-PA or thrombectomy (14.7 months). However, differences were not statistically significant (*p* > 0.05). A total of 21 patients required repeat interventions, with an average patency duration of 25.13 months after reintervention. **Conclusions:** This study suggests that endovascular treatment, especially balloon angioplasty, plays a key role in maintaining fistula patency.

## 1. Introduction

Chronic kidney disease (CKD) affects approximately 850 million people worldwide, with approximately 4 million requiring renal replacement therapy. Even among transplant patients, some patients with non-functioning kidney transplants also require hemodialysis, highlighting the continuous and increasing demand for dialysis each year [1]. According to the Kidney Disease Outcomes Quality Initiative (KDOQI) guidelines, native arteriovenous fistula (AVF) remains the preferred vascular access for long-term hemodialysis [2]. AVFs have been considered the gold standard for more than fifty years due to superior patency and low complication rates compared to alternative access methods, such as arteriovenous grafts and tunneled or non-tunneled catheters [3]. Underlining the critical role of AVFs in renal replacement therapy, reliable vascular access remains essential for effective hemodialysis [4]. Extensive research and clinical guidelines have demonstrated that AV fistulas are the preferred method for hemodialysis, offering superior outcomes with fewer complications [5].

Cephalic and basilic veins are commonly preferred for the creation of AVFs, owing to their accessibility and favorable anatomical characteristics. These are medium-sized veins, typically measuring between 1 and 5 mm in diameter, and are characterized by a thin intimal layer that facilitates surgical manipulation and hemodynamic adaptation [6]. Despite the recognized advantages of native AVFs—such as lower infection rates and longer potential patency compared to synthetic grafts—achieving and maintaining long-term patency continues to pose a significant clinical challenge. Studies indicate that nearly 50% of AVFs ultimately fail due to various pathophysiological mechanisms. A key contributor to AVF dysfunction is juxta-anastomotic endothelial injury, which triggers intimal hyperplasia and leads to progressive vascular stenosis [7,8]. One of the main sources of this endothelial trauma is the repeated cannulation of the AVF site during hemodialysis sessions. These repetitive punctures exacerbate local vascular injury and promote a cascade of inflammatory and proliferative responses. Consequently, abnormal flow dynamics—such as turbulence and blood flow stasis—develop within the fistula, creating a pro-thrombotic milieu that significantly heightens the risk of AVF thrombosis and occlusion [9,10].

To address these complications, advancements in endovascular interventions have significantly improved the long-term viability of AVFs. Techniques such as percutaneous transluminal angioplasty (PTA), thrombolysis, and thromboaspiration have been shown to restore patency and extend the usability of AVFs for hemodialysis [11,12]. These minimally invasive procedures play a crucial role in maintaining vascular access, reducing the need for alternative access methods, and ultimately enhancing the quality of care for hemodialysis-dependent patients [12,13].

In this study, we aimed to present our single-center experience regarding the effectiveness of endovascular treatment in patients with hemodialysis fistula dysfunction. Additionally, we sought to evaluate the long-term follow-up outcomes of these patients and to investigate the impact of different treatment strategies on both primary and long-term patency rates.

## 2. Materials and Methods

**Figure 1:**
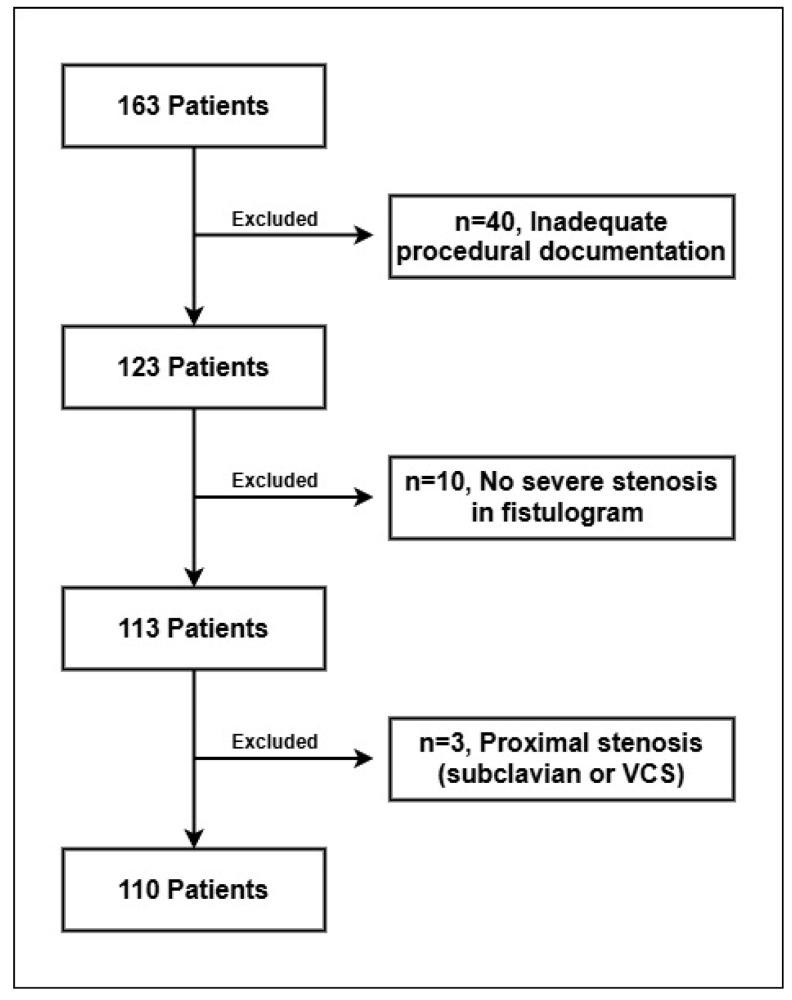
Exclusion criteria flowchart.

This retrospective study included patients who presented with hemodialysis fistula dysfunction to the Interventional Radiology Department of our institution between 2017 and 2022. An initial cohort of 163 patients was assessed. Exclusion criteria comprised patients who underwent imaging without demonstrable evidence of stenosis or thrombosis, those with central venous stenosis involving proximal veins such as the subclavian vein or superior vena cava, and cases with incomplete or inadequate procedural documentation. Following the application of these criteria, a total of 110 patients were deemed eligible and included in the final analysis (Figure 1).

Detailed demographic and procedural data were systematically collected. These included the anatomical location of the arteriovenous fistula, specific site of stenosis, presence or absence of thrombotic occlusion, performance of thrombectomy, administration of tissue plasminogen activator (t-PA), and implementation of balloon angioplasty. Furthermore, data regarding primary patency status, duration of post-intervention fistula patency, requirement for repeat endovascular interventions, and mean patency duration following both initial and subsequent procedures were meticulously recorded for outcome assessment.

### 2.1. Procedure Details

Patients presenting with hemodialysis fistula dysfunction were initially evaluated using Doppler ultrasound in the Interventional Radiology Unit. The assessment focused on identifying the presence of stenosis or thrombosis, with particular attention given to the anastomosis, juxta-anastomotic segment, and the draining venous pathway. If a vascular lesion resulting in greater than 50% luminal narrowing was detected, the patient was considered eligible for endovascular intervention. Depending on the nature and extent of the pathology, one of three treatment strategies was selected: thrombolytic therapy with t-PA and/or thrombectomy, balloon angioplasty, or a combination of both modalities.

A transvenous approach was generally preferred for vascular access, and a 5 French (5F) vascular sheath was inserted. A diagnostic fistulogram was then performed to accurately localize the site and severity of the stenotic lesion. The lesion was crossed using 0.035-inch or 0.018-inch hydrophilic guidewires in conjunction with dedicated support catheters. Balloon angioplasty was subsequently performed using balloons selected to match the vessel diameter. Balloons were typically inflated for between 2 and 5 min and maintained in position until adequate luminal expansion was achieved. Following dilation, a post-angioplasty fistulogram was obtained to evaluate the success of the intervention and confirm vessel patency. In cases of residual or recurrent stenosis, drug-coated balloon angioplasty was employed, and the procedure was repeated as needed.

For patients with isolated thrombosis, targeted thrombolysis with t-PA or mechanical thrombectomy was conducted under ultrasound guidance. The choice between these two treatment modalities was based on thrombus age. Thrombolytic therapy was initiated in cases with hyperacute thrombus, whereas mechanical thrombectomy was performed for subacute thrombus. In thrombolysis treatment, a selective intra-thrombus injection of 2 to 4 mg t-PA was administered, followed by a 30 min waiting period before re-imaging. Mechanical thrombectomy was added to thrombolytic therapy in patients who had more than 30% residual thrombus within the lumen following thrombolysis. Mechanical thrombectomy was also carried out using dedicated aspiration catheter systems, as appropriate. In addition, in cases where both significant stenosis and thrombotic occlusion were present, a combined therapeutic approach involving thrombolysis and angioplasty was utilized to restore adequate fistula function. All procedures were performed by an interventional radiologist with 10 years of experience.

### 2.2. Procedure Outcome and Follow-Up

Initially, the primary patency status of each patient was evaluated. Primary patency was defined as a successful reduction in the stenosis to less than 30% luminal narrowing following the intervention. Cases in which post-procedural residual stenosis exceeded 30% were categorized as procedural failures. All patients were scheduled for routine post-procedural follow-up. Fistula patency was systematically evaluated at the 3rd and 6th months after the intervention. Both grayscale ultrasound findings and color Doppler ultrasound findings (flow rate, flow direction, and flow characteristics) were used to determine whether patency persisted at follow-up imaging. Patients who were lost to follow-up or did not attend scheduled visits were excluded from the respective 3-month and 6-month patency analyses.

In addition to primary outcomes, long-term patency durations following the initial procedure were assessed. Moreover, the need for repeat endovascular interventions was recorded, and subsequent patency durations following these reinterventions were also analyzed to provide a comprehensive view of treatment durability.

### 2.3. Statistical Analysis

Statistical analyses were performed based on the distribution characteristics of the collected data and the nature of the comparisons. Continuous variables that did not follow a normal distribution were compared using the Mann–Whitney U test. Categorical variables were analyzed using either the Chi-squared test or Fisher’s exact test, depending on cell counts and expected frequencies. For comparisons involving three or more independent groups, One-Way Analysis of Variance (ANOVA) was employed to assess differences. To further evaluate factors associated with primary and secondary patency outcomes, odds ratios (ORs) were calculated with corresponding confidence intervals. A *p*-value of less than 0.05 was considered indicative of statistical significance. All statistical analyses were conducted using SPSS software (version 23.0; IBM Corp., Armonk, NY, USA).

## 3. Results

A total of 110 patients (81 males and 29 females) with hemodialysis fistula dysfunction were retrospectively analyzed. The mean age was 61.93 years in the patency group and 60 years in the non-patency group, with no statistically significant difference between the two (*p* = 0.9875). The majority of fistulas were located at the radiocephalic site (60%), followed by brachiocephalic (36%) and brachiobasilic (4%) locations. No significant association was found between fistula location and primary patency outcomes (*p* = 0.5969). Stenosis was located in the juxta-anastomotic region of 59 patients and in the venous side of 51 patients. Thrombus was present in 67 patients, whereas 43 patients had no evidence of thrombosis. There was no statistically significant association between the localization of stenosis or the presence of thrombosis and primary patency outcomes (Table 1).

### 3.1. Primary Patency Outcomes

**Figure 2:**
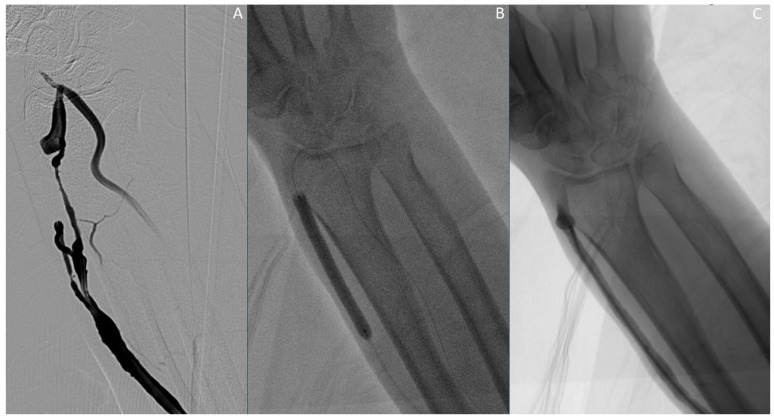
Distal radiocephalic fistula. (**A**) Severe stenosis is observed in the fistula vein. (**B**) Balloon angioplasty is performed at the level of the stenosis. (**C**) Post-angioplasty imaging shows adequate luminal patency at the site of the venous stenosis.

Out of the 110 patients included in the study, 100 patients (90.9%) achieved primary patency, while 10 patients (9.1%) experienced primary patency failure. Figure 2 illustrates the angiographic findings of a representative patient in whom primary patency was successfully achieved following endovascular treatment. Among the treatment modalities, balloon angioplasty was significantly associated with favorable patency outcomes (*p* = 0.0077). In contrast, the use of tissue plasminogen activator (tPA), mechanical thrombectomy, and drug-coated balloon angioplasty did not demonstrate a statistically significant impact on primary patency rates (*p* > 0.05) (Table 2).

### 3.2. 3-Month and 6-Month Follow-Up

At 3- and 6-months, 22 patients were lost to follow-up. Therefore, the analysis was performed on the remaining 88 patients for whom follow-up data were available. At the 3-month follow-up (n = 88), primary patency was preserved in 73 patients (83.0%), while 15 patients (17.0%) experienced loss of patency. Among the evaluated treatment modalities, the use of drug-coated balloon angioplasty yielded the highest odds ratio for patency maintenance (OR = 4.25); however, this association did not reach statistical significance (*p* = 0.2889).

At the 6-month follow-up, 64 patients (72.7%) retained primary patency. In this interval, conventional balloon angioplasty was associated with the highest odds of continued patency (OR = 3.06); however, this association did not reach the threshold for statistical significance (*p* = 0.0662) (Table 3).

### 3.3. Comparative Analysis Between Treatment Groups

ANOVA analysis revealed no statistically significant difference between the three treatment groups (balloon angioplasty, t-PA and/or thrombectomy, and combined treatment) in long-term mean fistula patency time (*p* = 0.322). Direct comparisons between the balloon angioplasty group and the non-balloon group (*p* = 0.124), the balloon group and the combination therapy group (*p* = 0.512), and the non-balloon group and combination therapy group (*p* = 0.335) did not yield significant differences in outcomes (Table 4).

### 3.4. Long-Term Follow-Up

**Figure 3:**
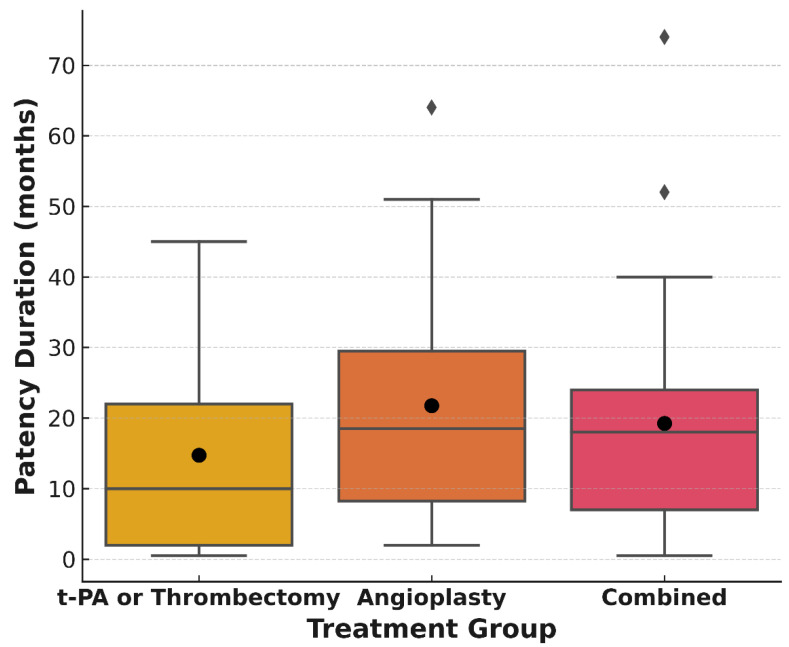
Box plot showing the distribution of post-intervention fistula patency durations across three treatment groups: t-PA and/or Thrombectomy, Angioplasty, and Combined. Black circles indicate the mean patency duration for each group. While angioplasty alone was associated with the highest average patency, combined treatment also yielded favorable outcomes in patients with more complex lesion profiles.

The mean fistula patency time among the 88 patients included in the follow-up was 19.45 months. The mean fistula patency time was 14.7 months in 17 patients treated with t-PA or thrombectomy without balloon angioplasty and 21.8 months in 38 patients treated with balloon angioplasty alone. The mean fistula patency time was 19.2 months in 33 patients with both treatment approaches. There was no statistically significant difference between the patency times of the three different treatment groups (*p* > 0.05). In Figure 3, the median and mean values of the long-term patency results according to the three treatment groups are given as box plots. A total of 21 patients required repeated procedures after the first procedure, and the mean fistula patency time of the patients who could be followed up in this group was 25.13 months.

## 4. Discussion

This study evaluated the effectiveness of endovascular treatment in patients with dysfunctional native hemodialysis AVFs. Primary patency was achieved in 90.9% of cases following intervention, indicating a high technical success rate. Among treatment modalities, only balloon angioplasty was significantly associated with improved patency outcomes (*p* = 0.0077), whereas tPA administration, thrombectomy, and drug-coated balloon angioplasty did not show significant benefits. At 3- and 6-months follow-up, patency rates were 83% and 72.7%, respectively. Although drug-coated balloons and standard angioplasty showed favorable odds ratios for patency maintenance, statistical significance was not reached. The mean post-procedural patency duration among followed patients was 19.45 months, suggesting that endovascular therapy provides durable outcomes in most cases.

Hemodialysis fistulas offer advantages over catheters in patients with chronic kidney disease (CKD), including better long-term patency, lower infection risk, and reduced morbidity rates [14]. However, despite these advantages, fistulas are frequently compromised by complications such as thrombosis and stenosis, which necessitate prompt and effective intervention. Endovascular treatment has emerged as a highly effective and minimally invasive option, demonstrating a high technical success rate and excellent clinical outcomes in restoring and maintaining fistula function. Techniques such as mechanical thrombectomy, catheter-directed thrombolysis using t-PA, conventional balloon angioplasty, and drug-coated balloon interventions have been shown to significantly contribute to prolonged patency and reduced need for surgical revision [15,16,17].

Recent advances in imaging guidance and catheter technology have further improved the precision and efficacy of these procedures. Several studies in the literature have investigated the clinical outcomes of endovascular treatment in this setting, reporting consistently favorable primary patency rates at 3–6 months post-intervention, with outcomes generally considered clinically satisfactory across various patient populations and treatment techniques [17,18,19]. Additionally, endovascular approaches allow for repeated interventions, which is particularly important given the chronic and progressive nature of AVF dysfunction in the hemodialysis population.

Thakker et al. evaluated 46 patients and reported the highest success rates in radiocephalic fistulas with juxta-anastomotic stenosis, achieving a technical success rate of 89.13% and a primary patency rate of 78.26% [20]. Additionally, the authors observed higher technical success in patients who underwent percutaneous PTA alone compared to those who received PTA combined with thrombectomy (88.23% vs. 84.2%). At the 3-month follow-up, patency was 82.35% in the angioplasty group and 73.7% in the combination therapy group. In comparison, our study, which included a larger cohort of 110 patients, demonstrated a slightly higher primary patency rate (90.9%). Notably, patients who underwent balloon angioplasty alone had significantly higher primary patency rates (*p* = 0.0077). However, no statistically significant difference was observed between the angioplasty and combination therapy groups regarding long-term patency (*p* = 0.512). Additionally, the 3-month patency rate in our study (83%) was comparable to that reported by Thakker et al.

Hendawy et al. reported a technical success rate of 88.3% in their study analyzing endovascular treatment in 60 patients [21]. The reasons for treatment failure included total occlusion, persistent false passage, resistant non-dilatable lesions, and high-origin radial artery. The primary patency rates at 3 and 6 months were 82% and 58%, respectively. While our study showed a similar 3-month patency rate (83%), the 6-month patency rate was notably higher (72.7%). Vignesh et al. evaluated 23 patients, initially treating them with PTA and performing thromboaspiration if necessary [22]. Their study reported technical and clinical success rates of 88.8% and 81.5%, respectively. With a mean follow-up of 9.5 months, eight patients experienced re-occlusion after initial patency, and four required repeat procedures (mean time to re-intervention: 5.5 ± 1.3 months). The primary patency rates at 3 and 6 months were 79% and 60%, respectively. In our study, the mean fistula patency duration was 14.7 months in the t-PA and/or thrombectomy group, 21.8 months in the balloon angioplasty group, and 19.2 months in the combined treatment group. Additionally, 21 patients required repeated interventions, and the mean fistula patency duration in this subgroup was 25.13 months.

In another study, Xinyan Hu et al. analyzed 114 endovascular procedures performed in 80 patients, comparing those with stenotic lesions (n = 51) and those with thrombosed fistulas (n = 63) [23]. Anatomical success rates were reported as 94% in the stenosis group and 89% in the thrombosis group. Clinical primary patency was achieved in 100% of patients in both groups following the initial intervention. At 3 months, primary patency was reported as 96% in the stenosis group and 87% in the thrombosis group, declining to 69% and 54%, respectively, by the 6-month follow-up. Unlike the study by Xinyan Hu et al., which stratified patients into distinct stenotic and thrombosed groups, our study did not categorize patients solely based on the presence or absence of thrombosis. Instead, we evaluated the effectiveness of endovascular treatment in a more integrated manner, reflecting real-world clinical practice where mixed pathologies are frequently encountered. In our cohort, treatment approaches were tailored according to the underlying pathology and included standalone or combined modalities such as balloon angioplasty, thrombectomy, and t-PA administration. This comprehensive approach allowed for the management of both stenotic and thrombotic components when present, without isolating them into mutually exclusive groups. Therefore, while their study offers insight into outcome differences between lesion types, our results provide a broader perspective on endovascular management in patients with heterogeneous AVF dysfunction.

In a prospective randomized study, Lookstein et al. compared drug-coated balloon angioplasty with standard balloon angioplasty in 330 patients [24]. Among them, 170 were treated with drug-coated balloons, and 160 received standard balloon treatment for target stenosis. At 6 months, the drug-coated balloon showed a significantly higher patency rate compared to the standard balloon (82.2% vs. 59.5%). Although our study did not directly compare drug-coated and standard balloon angioplasty, it can be suggested that drug-coated balloons may be considered in cases where standard balloon angioplasty is insufficient.

This study has several limitations. First, it is a single-center retrospective study. Second, some patients were excluded due to missing follow-up data. Third, the treatment groups had an inhomogeneous distribution. Fourth, some patients had short follow-up durations. Future prospective randomized studies with larger and more evenly distributed patient groups could better validate the effectiveness of endovascular treatment.

## 5. Conclusions

This study highlights the high effectiveness of endovascular treatment in the management of dysfunctional native hemodialysis AVFs, with a notable primary patency rate of 90.9% following intervention. Patency was favorably sustained in the majority of patients, with rates of 83% and 72.7% at 3 and 6 months, respectively. These findings underscore the pivotal role of endovascular therapy as a first-line treatment strategy, offering not only high technical success but also durable clinical outcomes. By preserving AVF function and minimizing the need for catheter dependence, endovascular interventions contribute significantly to improving the quality of care and long-term prognosis in patients undergoing hemodialysis.

## Figures and Tables

**Table 1:** Patient characteristics and analysis of age, fistula locations, stenosis locations, and thrombus status.

Variables	Cathegories	Patency (n = 100)	Non-Patency (n = 10)	*p*-Value
Age		61.93	60	0.9875
Left-right arm	Left	84	6	0.1481
	Rigt	16	4	
Fistula Locations	Brachiobasilic	4	0	0.5969
	Brachiocephalic	36	5	
	Radiocephalic	60	5	
Stenosis Locations	Juxta-anastomotic	54	5	1.0
	Vein	46	5	
Thrombus	Yes	58	9	0.1
	No	42	1	

**Table 2:** Association of different treatment types with primary patency.

		Primary Patency (n = 110)		*p*-Value
		Yes (n = 100)	No (n = 10)	
tPA	Yes	53 47	5 5	1.0
	No			
Thrombectomy	Yes	33 67	4 6	0.9237
	No			
Balloon angioplasty	Yes	82 18	4 6	0.0077
	No			
Drug-coated balloon angioplasty	Yes	20 80	0 10	0.2570
	No			

tPA: tissue plasminogen activator.

**Table 3:** Three- and six-month patency analysis and effect of treatment types.

		3-Month Patency (n = 88)		*p*-Value	OR	6-Month Patency (n = 88)		*p*-Value	**OR**
		Yes (n = 73)	No (n = 15)			Yes (n = 64)	No (n = 24)		
tPA	Yes	36 37	11 4	0.1539	0.35	32 32	15 9	0.3434	0.60
	No								
Thrombectomy	Yes	24 49	8 7	0.1539	0.43	21 43	11 13	0.3216	0.58
	No								
Balloon angioplasty	Yes	61 12	10 5	0.1556	2.54	55 9	16 8	0.0662	3.06
	No								
Drug-coated balloon angioplasty	Yes	17 56	1 14	0.2889	4.25	15 49	3 21	0.3761	2.14
	No								

tPA: tissue plasminogen activator, OR: odds ratio.

**Table 4:** Statistical analysis of different treatment types in long-term fistula patency.

Treatment Cohorts	*p*-Value
Balloon angioplasty vs. non-balloon angioplasty	0.124
Balloon angioplasty vs. combined treatment	0.512
Non-balloon angioplasty vs. combined treatment	0.335

## Data Availability

The data supporting this study’s findings are available from the corresponding author upon request.
